# Differential long non-coding RNA expression profile and function analysis in primary Sjogren’s syndrome

**DOI:** 10.1186/s12865-021-00439-3

**Published:** 2021-07-20

**Authors:** Xiaochan Chen, Qi Cheng, Yan Du, Lei Liu, Huaxiang Wu

**Affiliations:** Department of Rheumatology, The Second Affiliated Hospital of Zhejiang University School of Medicine, No.88 Jiefang Road, Hangzhou, 310009 China

**Keywords:** LncRNA, GABPB1-AS1, Sjogren’s syndrome, PBMC

## Abstract

**Background:**

Primary Sjögren’s syndrome (pSS) is a chronic autoimmune disease characterized by abnormal immune cell activation. This study aimed to investigate differentially expressed long non-coding RNA (lncRNA) in peripheral blood mononuclear cells (PBMCs) in patients with pSS to identify lncRNAs that affect pSS pathogenesis.

**Methods:**

Total RNA was extrated from PBMCs of 30 patients with pSS and 15 healthy persons. Transcriptome sequencing was used to screen differentially expressed lncRNAs and mRNAs in 8 RNA samples from the discovery cohort. The differentially expressed mRNAs underwent functional enrichment analysis. A protein interaction relationship (PPI) and competitive endogenous RNA (ceRNA) network was constructed. Real-time PCR was used to validate screened lncRNAs in all 45 RNA samples..

**Results:**

1180 lncRNAs and 640 mRNAs were differentially expressed in pSS patients (fold change > 2 in healthy persons). The PPI network was constructed with 640 mRNAs and a ceRNA network with four key lncRNAs (GABPB1-AS1, PSMA3-AS1, LINC00847 and SNHG1). Real-time PCR revealed that GABPB1-AS1 and PSMA3-AS1 were significantly up-regulated 3.0- and 1.4-fold in the pSS group, respectively. The GABPB1-AS1 expression level was positively correlated with the percentage of B cells and IgG levels.

**Conclusions:**

GABPB1-AS1 was significently up-regulated in pSS patients, and its expression level is positively correlated with the percentage of B cells and IgG levels. GABPB1-AS1 may be involved in the pathogenesis of pSS and may be a promising biological marker.

**Supplementary Information:**

The online version contains supplementary material available at 10.1186/s12865-021-00439-3.

## Background

Primary Sjogren’s syndrome (pSS) is a chronic autoimmune disease which clinically presents with endocrine gland dysfunction, mainly occurring in the salivary and lacrimal glands. Histopathology typically shows infiltration of lymphocytes. In particular, large amounts of activated B lymphocytes are observed, which produce a variety of autoantibodies and cytokines; this is related to the occurrence and development of the disease. Although multiple factors such as genetic and environmental factors are thought to be related to this abnormal cellular activity, the specific mechanisms of pSS have not been fully elucidated.

Non-coding RNAs are RNA sequences in the human genome that cannot encode proteins and are largely categorized into short-chain non-coding RNAs (microRNAs, miRNAs) (< 200 nt) and long-chain non-coding RNAs (lncRNAs) (> 200 nt). A large number of studies have shown that miRNAs participate in regulating the pathogenesis of some autoimmune diseases due to their control of gene expression [1, 2]. Moreover, miRNAs affect the development of Sjögren’ s syndrome [3]. Unlike miRNAs, the functions of most lncRNAs are largely unknown because their structure and regulatory patterns are more complex than miRNAs. The known lncRNAs appear to exert a regulatory role in gene transcription or expression and function as scaffold molecules to regulate the expression of certain miRNA target genes [4]. Therefore, lncRNAs may affect the development of autoimmune diseases by regulating gene expression during immune cell differentiation and immune responses.

Some preliminary studies have found the role of lncRNAs in regulating gene expression, consequently affecting the pathogenesis of immune diseases. For example, a study showed that lncRNA NeST affects microbial susceptibility by increasing interferon-γ (IFN-γ) levels in activated CD8(+) T cells [5]. lncRNA NEAT1 regulates chemokine and cytokine expression and contributes to the progression of systemic lupus erythematosus [6]. lncRNA Hotair expression in differentiated osteoclasts and rheumatoid synovial cells is closely related to a significant increase in MMP-2 and MMP-13 levels, suggesting that lncRNA Hotair participates in rheumatoid arthritis by promoting matrix metalloproteinase (MMP) production [7]. lncRNA FIRRE regulates the expression of VCAM1, IL-12p40, and other inflammatory genes in the innate immune system at the post-transcriptional level [8]. It can promote B cell proliferation by activating the Wnt/β-catenin pathway and it is related to the pathogenesis of diffuse large B cell lymphoma [9]. Based on the previous studies, lncRNAs are expected to be biomarkers for disease diagnosis or therapeutic targets.

Recently, two researches have focused on the expression profile of LncRNAs in peripheral blood mononuclear cells (PBMCs) of pSS patients. Peng et al. found two significantly up-regulated lncRNAs (NRIR and BISPR) and discovered they may regulate important genes involved in the Type I interferon signaling pathway [10]. Dolcino et al. observed six DE lncRNAs (CTD-2020 K17.1, LINC00657, RP11-169 K16.9, LINC00511, RP11-372 K14.2 and RP11-214O1.2) and found they target gene pathways that are involved in epithelial cell damage, autoimmunity and B cell hyperactivation [11]. To date, however, the correlation between lncRNAs and pSS has not been well studied.

In order to provide more reliable evidences, this study analyzed lncRNA and mRNA expression profiles in PBMCs of patients with pSS using transcription sequencing and then identified lncRNAs related to pSS using real-time PCR in a relatively large cohort. Our findings provide a new perspective for the pathogenesis study and identified potential disease-related biological markers and therapeutic targets for pSS.

## Result

### Patient characteristics

The characteristics of the 30 pSS patients are shown in Table 1. The enrolled patients were positive for antinuclear antibodies (≥1:80), in which 25 and 8 patients were positive for anti-SSA/Ro antibodies and anti-SSB/La antibodies, respectively. Ocular and oral dryness were reported in approximately 20 and 24 patients, respectively. Lymphocyte infiltration was found in 25 patients who underwent labial salivary gland biopsies. The median Eular Sjogren’s syndrome disease activity index (ESSDAI) score was 3.8, the median B cell/lymphocyte was 13.4%, and the mean IgG was 16.3 ± 4.3. g/L. B cell lymphoma occurred in one patient after 3 months in the study.

**Table 1:** The detailed demographic, clinical, and laboratory characteristics of 30 pSS patients

Indexes	RNA sequencing(*n* = 4)	PCR validation(*n* = 30)
Sex, no. Male/female	0/4	0/30
Age, mean (SD) years	47.0(12.1)	52.7 (13.1)
Disease duration, median (IQR) years	3.5(1.0)	1.0(6.2)
Oral dryness (VAS,1–10), median (IQR)	5.0(2.0)	5.5(4.3)
Ocular dryness (VAS,1–10), median (IQR)	3.5(1.0)	3.0(5.3)
Grading of labial salivary gland biopsies, no.		
Grade2, no.	0	1
Grade3, no.	2	11
Grade4, no.	2	13
IgG (g/L), mean ± SD	14.9(2.5)	17.5(3.4)
C3 (g/L), mean ± SD	0.89(0.24)	0.83(0.24)
C4 (g/L), mean ± SD	154.3(48.1)	156.8(72.6)
ESR (mm/h), mean ± SD	25(19)	30(45)
RF (IU/mL), mean ± SD	12.4(2.7)	12.7(19.8)
SSA, no. +/−	4/0	25/5
SSB, no. +/−	1/3	8/22
B cell, %. median (IQR)	23.1(8.3)	18.2(7.8)
ESSDAI score, median (IQR)	2.5(1.0)	3.8(1.9)

*SD* standard deviation, *IQR* inter-quartile range, *VAS* visual analogue scale, *IgG* immunoglobulin G, *C3/C4* complement 3/4, *ESR* erythrocyte sedimentation rate, *RF* rheumatoid factor, *ESSDAI* Eular Sjogren’s syndrome disease activity index

### High-throughput lncRNA and mRNA expression profile in PBMC

**Fig. 1:**
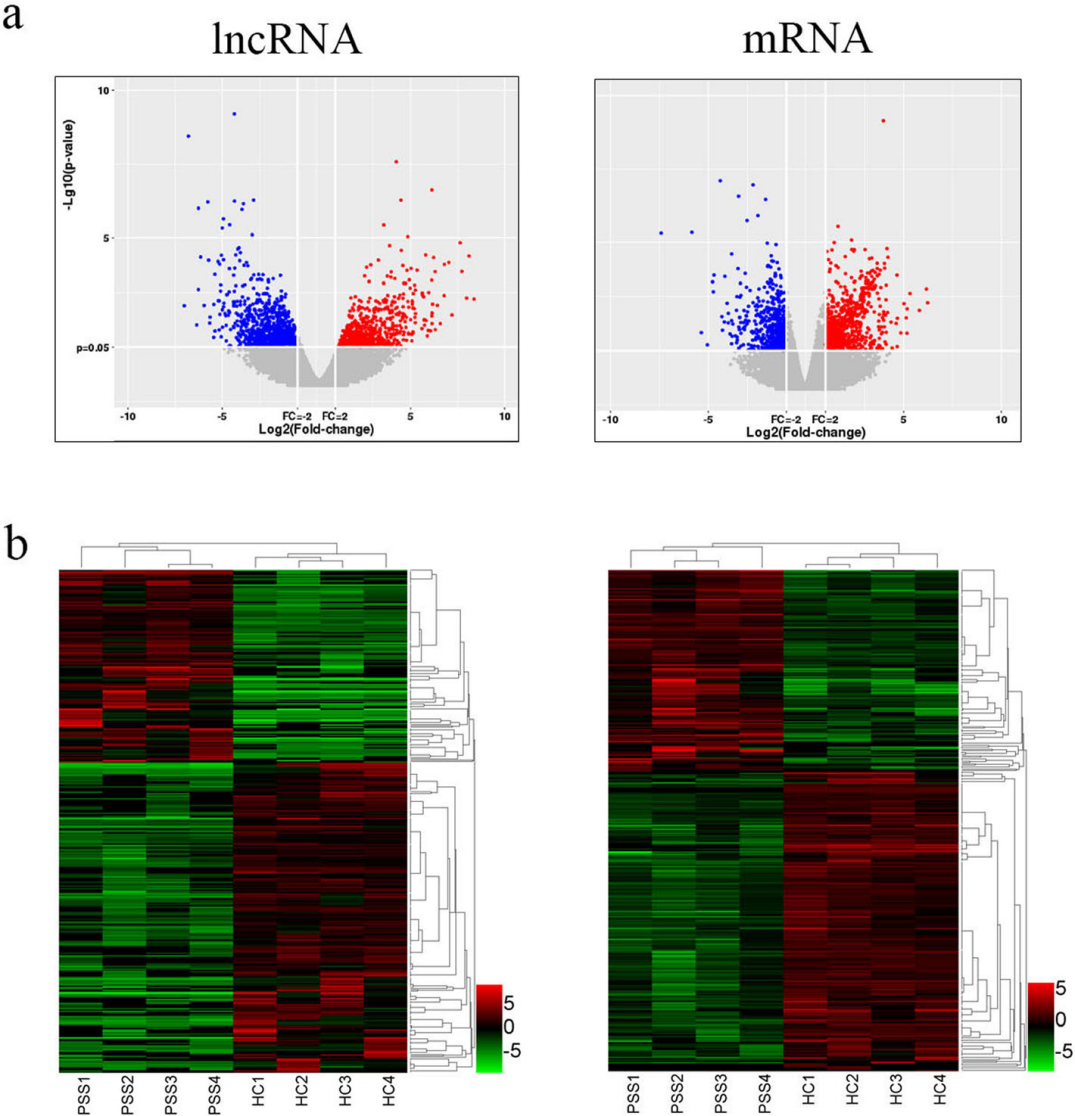
Expression profile of LncRNAs and mRNAs in pSS patients. PBMCs of pSS patients and healthy persons were collected for transcriptome sequencing analysis. The differentially expressed lncRNAs and mRNAs were illustrated using (a) volcanic map and (b) heatmap. Up-regulation is represented in red. Down-regulation is represented in blue in the volcanic map and green in the heatmap

We found 1180 lncRNAs and 640 mRNAs with significantly different expression in the PBMC of pSS patients based on transcriptome sequencing (fold change > 2, *p* < 0.05). The up-regulated genes included 497 lncRNAs and 256 mRNAs. The down-regulated genes included 683 lncRNAs and 384 mRNAs. The differentially expressed (DE) genes are displayed using a volcanic map (Fig. 1a) and heatmap (Fig. 1b). Tables 2 and 3 list the 15 most up-regulated and down-regulated DE lncRNAs and DE mRNAs.

**Table 2:** Top 15 significantly differential expressed lncRNAs between pSS patients and control subjects

up-regulated lncRNA	***P*** value	Fold change	down-regulated lncRNA	***P*** value	Fold change
GABPB1-AS1	1.36E-09	738.802	RP11-343H5.4	2.17E-14	− 856.93
lnc-AC012652.1.1–1-11	1.36E-05	289.846	m121218_154352_00126_c100	1.18E-06	−30.374
lnc-TMEM65–4-1	1.71E-08	51.288	brain_Pred31224	2.85E-08	−26.599
lnc-RBPMS2–3-1_dup1	4.34E-04	31.393	lnc-CNOT7–2-1	3.41E-04	−10.070
m121212_224522_00126_c100	1.15E-08	18.981	lnc-ZNF507–1-1_dup1	9.88E-05	−8.296
lnc-YDJC-1-2_dup1	3.99E-03	8.583	LINC00847	8.57E-03	−7.030
m130128_030356_00126_c100	1.81E-03	8.125	m121218_132759_00126_c100	2.00E-06	−6.950
NRIR	2.07E-03	7.079	lnc-MYOM2–7-1_dup1	3.69E-04	−5.980
PSMA3-AS1	4.37E-04	5.963	m121218_180114_00126_c100	3.61E-03	−5.788
lnc-ZNF503-AS2–4-1	5.88E-03	4.110	CUFF.400492	1.25E-03	−5.056
lnc-C3orf67–3-1_dup1	6.69E-03	3.961	lnc-SART3–2-1_dup1	2.37E-03	−4.750
lnc-STRBP-9-1	8.99E-03	3.689	brain_Pred8666	7.42E-03	−4.069
RPL32P1	7.02E-04	3.567	SNHG1	2.31E-04	−3.550
lnc-RNF150–2-4	1.25E-02	3.496	lnc-CLEC4C-1-1_dup1	2.86E-03	−3.498
lnc-ERMN-2-1_dup1	8.88E-03	3.104	EBLN3	6.98E-03	−3.423

**Table 3:** Top 15 significantly differential expressed mRNAs between pSS patients and control subjects

up-regulated mRNA	***P*** value	Fold change	down-regulated mRNA	***P*** value	Fold change
MPO	7.03E-05	7.779	MYOM2	1.68E-07	−9.794
SIGLEC1	7.42E-08	5.642	SH2D1B	1.72E-06	−4.162
IFI44L	1.16E-06	5.570	COL6A2	1.68E-06	−4.160
IFI44	9.43E-08	3.296	KIR2DL3	8.09E-06	−3.965
IFI6	1.45E-06	2.868	ARMCX3	3.29E-08	−3.629
PCNP	1.96E-06	2.840	KLRF1	2.63E-05	−3.585
CMPK2	3.75E-05	2.590	GNLY	6.24E-05	−3.580
EPSTI1	1.42E-07	2.559	PRF1	1.75E-05	−3.406
CD83	3.75E-07	2.557	APEX1	8.96E-06	−3.370
ZNF267	1.02E-05	2.525	SPON2	8.17E-05	−3.291
PIM3	1.12E-04	2.460	FGFBP2	1.15E-04	−3.192
U2AF1	3.65E-05	2.313	KLRB1	1.13E-05	−2.793
SPI1	9.36E-06	2.290	CPA3	9.46E-05	−2.754
PLSCR1	2.90E-06	2.282	IL2RB	4.00E-05	−2.471
LAP3	4.52E-06	2.089	LDLRAP1	5.30E-05	−2.301

### Functional prediction and protein interaction relationship (PPI) network of DE mRNAs

The 640 DE mRNAs were subjected to GO and KEGG pathway detection to determine the correlation of signaling pathways to pSS pathological mechanisms. Table 4 lists the top 30 significant GO enrichment of differentially expressed mRNAs. Significantly enriched biological processes known to affect pSS include immune response, chemokine-mediated signaling pathway, cell adhesion, type I interferon signaling pathway, and inflammatory response. Significantly enriched cellular components include the plasma membrane, extracellular region, integral component of membrane, and extracellular exosome. Significantly enriched molecular functions include calcium ion binding, protein homodimerization activity, receptor activity, and heparin binding. Significantly enriched pathways include chemokine signaling pathway, PI3K-Akt signaling pathway, transcriptional misregulation in cancer, natural killer cell-mediated cytotoxicity, and cytokine-cytokine receptor interaction (Table 5).

**Table 4:** The Top 30 of significant GO enrichment (biological process, molecular function, and cellular component) of differential expressed mRNAs

GO enrichment	Fold enrichment	Gene number	***P*** value	GO domain
cellular ion homeostasis	18.832	3	0.009	Biological process
branching involved in labyrinthine layer morphogenesis	13.950	4	0.002	
response to yeast	12.072	5	0.001	
activation of transmembrane receptor protein tyrosine kinase activity	10.462	4	0.006	
labyrinthine layer blood vessel development	8.260	5	0.003	
positive regulation of cytokine production	7.847	6	0.001	
negative regulation of viral genome replication	7.062	9	<0.001	
chemokine-mediated signaling pathway	6.631	15	<0.001	
regulation of angiogenesis	6.075	6	0.003	
positive regulation of peptidyl-threonine phosphorylation	5.812	5	0.010	
defense response to fungus	5.812	5	0.010	
response to cold	5.231	6	0.005	
cell maturation	5.231	6	0.005	
cellular defense response	5.062	10	<0.001	
positive regulation of angiogenesis	4.913	18	<0.001	
calcium-mediated signaling	4.308	7	0.005	
wound healing	3.923	10	0.001	
type I interferon signaling pathway	3.923	8	0.004	
positive regulation of protein kinase B signaling	3.737	10	0.001	
cellular response to interleukin-1	3.537	8	0.007	
immune response	2.908	39	<0.001	
defense response to virus	2.853	15	0.001	
chemotaxis	2.830	11	0.006	
azurophil granule	11.667	4	0.004	Cellular component
collagen trimer	3.487	10	0.002	
chemokine receptor activity	9.350	5	0.002	Molecular function
growth factor binding	5.887	5	0.010	
chemokine activity	5.190	8	0.001	
heparin binding	3.576	18	<0.001	
receptor activity	2.930	20	<0.001

**Table 5:** Significantly enriched pathways of differential expressed mRNAs

Kegg pathway enrichment	Fold enrichment	Gene number	***P*** value
Malaria	4.044	26	1.14E-06
Cytokine-cytokine receptor interaction	3.029	15	0.002
Protein digestion and absorption	2.574	16	0.002
Transcriptional misregulation in cancer	2.543	7	0.007
Chemokine signaling pathway	2.435	10	0.028
Natural killer cell mediated cytotoxicity	2.320	14	0.034
Rap1 signaling pathway	1.887	20	0.034
PI3K-Akt signaling pathway	1.641	8	0.035

**Fig. 2:**
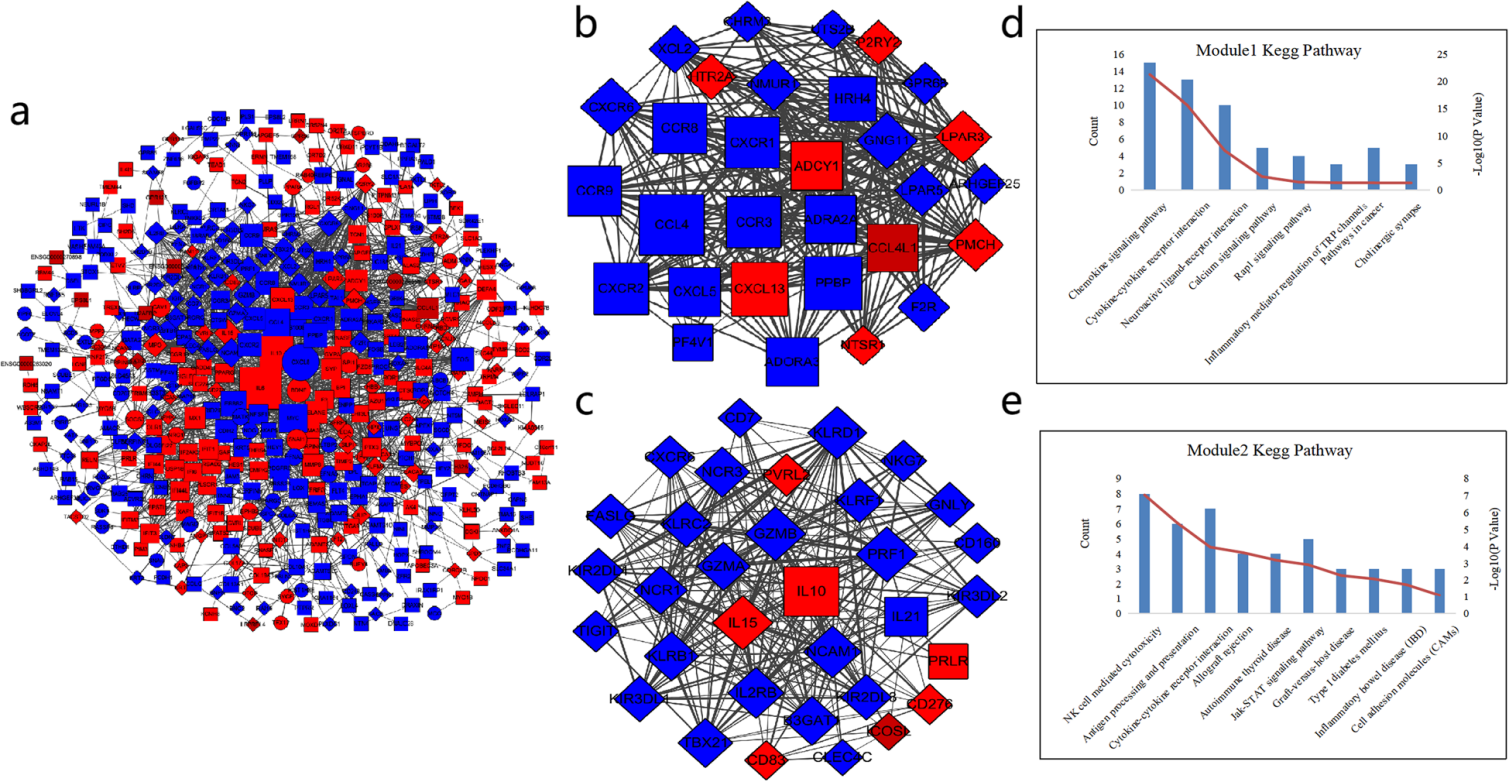
PPI network of DE mRNAs. (a) PPI network of 640 DE mRNAs, (b) functional module 1, (c) functional module 2. (d) Pathways identification in module 1 by KEGG pathway. (e) Pathways identification in module 2 by KEGG pathway. Red: up-regulated gene; blue: down-regulated gene. Rectangle: cluster gene; ellipse: outlier gene; diamond: overlap gene. The width of lines represents the combined_score of the two interacted proteins

According to the description in the Methods section, we obtained 2033 protein interaction pairs in 488 DE mRNAs, including 201 up-regulated genes and 287 down-regulated genes. The PPI network of 640 DE mRNAs was built (Fig. 2a), and two important functional modules were obtained. Table 6 shows the top 10 genes which resulted from the network connectivity analysis, among which IL-6, IL-10, and CXCL8 are at the center of the network and possibly the hub genes in the PPI network. Through module analysis, we obtained two important functional modules (Fig. 2b and c). Module 1 contains 30 genes and 292 interaction pairs, while module 2 contains 33 genes and 266 interaction pairs. The pathways that the genes in module 1 are involved in include the calcium signaling pathway, cytokine-cytokine receptor interaction, and chemokine signaling pathway (Fig. 2d). The pathways that the genes in module 2 are involved in include natural killer cell-mediated cytotoxicity, cytokine-cytokine receptor interaction, and JAK-STAT signaling pathway (Fig. 2e). These genes and pathways are known to influence the pathogenesis of pSS.

**Table 6:** Top10 in the result of PPI network connectivity analysis

Gene	Type	Degree
IL6	Up	90
CXCL8	Down	73
IL10	Up	64
NCAM1	Down	49
MYC	Down	46
IL15	Up	45
CCL4	Down	43
GZMB	Down	42
PRF1	Down	38
CXCR6	Down	38

*PPI* protein interaction relationship

### Competitive endogenous RNA (ceRNA) network of four selected lncRNAs

**Fig. 3:**
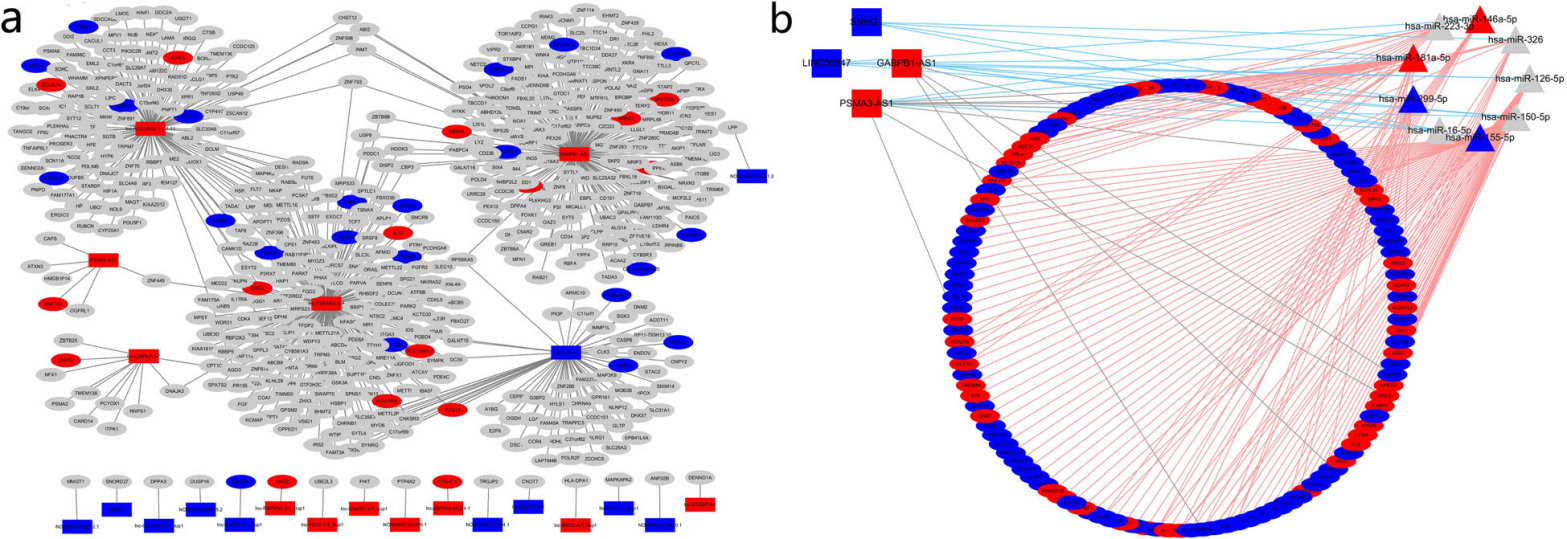
CeRNA network of selected lncRNAs. (a) The regulation network of the top 15 up-regulation and down-regulation DE lncRNAs predicted by Trans and Cis regulation. (b) CeRNA network of four key lncRNAs based on starBase. Red: up-regulated gene; blue: down-regulated gene. Rectangle: lncRNA; ellipse: mRNA; triangle: miRNA. Grey line: lncRNA-mRNA interaction; red line: miRNA-mRNA interaction; blue line: lncRNA-miRNA interaction

**Fig. 4:**
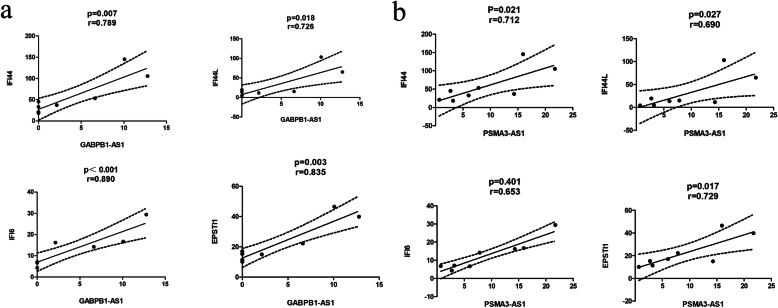
Correlation analysis between two up-regulated LncRNAs and four pSS related up-regulated mRNAs (IFI44, IFI44L, IFI6, EPSTI1). (a) GABPB1-AS1. (b)PSMA3-AS1

The regulation network of the top 15 up-regulation and down-regulation DE lncRNAs predicted by Trans and Cis regulation was constructed (Fig. 3a). Among them, GABPB1-AS1 regulates 187 genes, including two up-regulated genes (TRPM4 and SPATS2L) and two down-regulated genes (SCUBE1, CTD-2192 J16.20). To verify whether the DE lncRNAs were involved in the pathogenesis of pSS via interaction with miRNA, the top 15 of up-regulated and down-regulated DE lncRNAs were selected to construct the ceRNA network based on starBase. Only experimentally validated miRNA or mRNA targets annotated in the software were retained. Four lncRNAs, including GABPB1-AS1, PSMA3-AS1, LINC00847, and SNHG1, were obtained and their ceRNA network is shown in Fig. 3b. In this ceRNA network, there are 17 pairs of lncRNA-miRNA regulating relationships, 11 pairs of lncRNA-mRNA regulating relationships, and 155 pairs of miRNA-mRNA regulating relationships. Co-expression analysis was made between 4 key lncRNAs and 15 most up-regulated and down-regulated DE mRNAs (Table 7). Some of these significantly up-regulated mRNAs (IFI44, IFI44L, IFI6, EPSTI1) have been identified to be involved in the pathogenesis of pSS according to previous literatures [12–14]. Figure 4 shows the co-expression relationship between these hob genes and 2 up-regulated key lncRNAs (GABPB1-AS1, PSMA3-AS1).

**Table 7:** Co-expression analysis between 4 key lncRNAs and top15 significantly differentially expressed mRNAs

LncRNA name	Regulation	Correlated mRNA name	Pearson r	***P*** value
GABPB1-AS1	Up	SIGLEC1	0.803	0.016
		IFI44L	0.726	0.018
		IFI44	0.789	0.007
		IFI6	0.890	0.001
		PCNP	0.880	0.004
		EPSTI1	0.835	0.003
		CD83	0.739	0.036
		SPI1	0.728	0.041
		PLSCR1	0.823	0.012
		LAP3	0.749	0.032
PSMA3-AS1	Up	IFI44	0.712	0.021
		EPSTI1	0.729	0.017
		CD83	0.767	0.026
		ZNF267	0.854	0.007
		U2AF1	0.902	0.002
LINC00847	Down	MYOM2	0.746	0.033
		SH2D1B	0.739	0.036
		KIR2DL3	0.756	0.030
		ARMCX3	0.838	0.010
		CPA3	0.787	0.021
SNHG1	Down	MYOM2	0.902	0.002
		COL6A2	0.951	<0.001
		KIR2DL3	0.810	0.015
		ARMCX3	0.846	0.008
		APEX1	0.840	0.009
		CPA3	0.808	0.015
		IL2RB	0.761	0.028
		LDLRAP1	0.833	0.010

### Validation with real-time PCR and function analysis

**Fig. 5:**
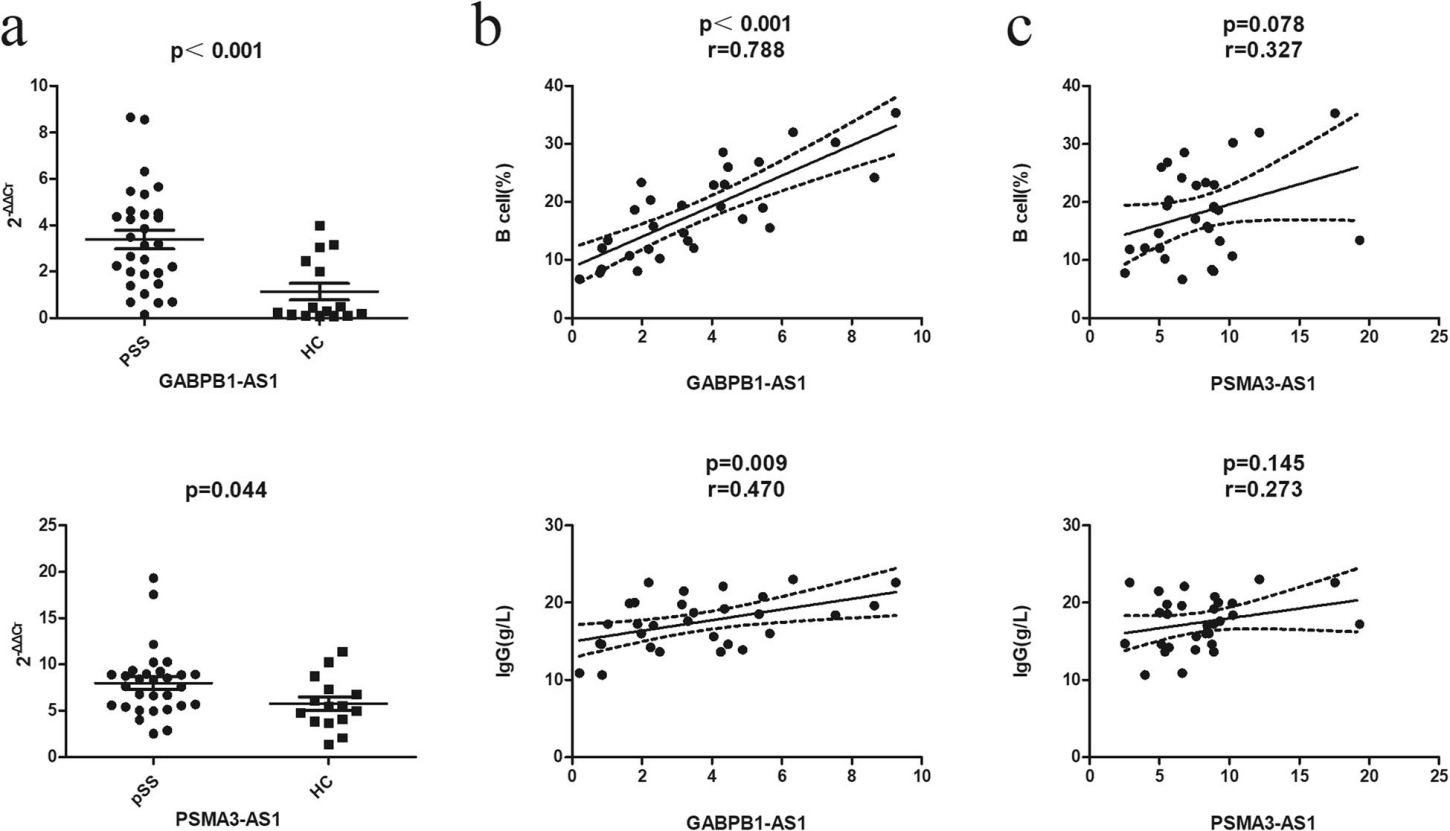
Correlation analysis between GABPB1-AS1 and PSMA3-AS1 and pathological factors. (a) GABPB1-AS1 and PSMA3-AS1 expression levels in pSS patients (*n* = 30) and healthy persons (*n* = 15) were determined using real-time PCR. (b) Correlation analysis between GABPB1-AS1 and B cell and IgG levels in pSS patients and health controls. (c) Correlation analysis between PSMA3-AS1 and B cell and IgG levels in pSS patients and health controls. The percentage of CD19+ B cells was detected by flow cytometry and IgG was measured by ELISA. The significant difference was *p* < 0.05

Two differentially expressed lncRNAs were chosen for further validation in an independent cohort including 30 patients with pSS and 15 healthy controls. The selected lncRNAs and their primers are presented in Table S2. The results demonstrate that GABPB1-AS1 and PSMA3-AS1 are significantly up-regulated 3.0- and 1.4-fold, respectively, in the pSS group compared to the healthy control (Fig. 5a). We further investigated the correlation of the two lncRNAs with clinical characteristics in patients with pSS and health controls. We found that the expression level of GABPB1-AS1 is positively correlated with the percentage of B cells and IgG levels (Fig. 5b). While the expression level of PSMA3-AS1 has no significant correlation with the percentage of B cells and IgG levels (Fig. 5c). Unfortunately, other clinical characteristics such as complement C3/C4, ESR, RF, SSA, and ESSDAI were not found any correlation with the expression level of GABPB1-AS1 or PASM3-AS1 (Table S3).

## Discussion

Recent evidence has demonstrated the essential role of lncRNAs in various types of autoimmune diseases including pSS due to their control of gene expression at multiple levels [15]. Shi et al. revealed eight significantly up-regulated lncRNAs in labial salivary glands in pSS patients and observed correlation between these lncRNAs and some clinical characteristics [16]. LncRNA TMEVPG1 and lncRNA PVT1 were found in another two studies to be up-regulated in CD4(+) T cells of pSS patients and were suggested to be involved in the pathogenesis of pSS [17]. However, the potential role of lncRNAs in pSS is still far from known. In this study, we payed attention to the expression profile of lncRNAs in PBMCs. We screened out DE mRNAs and DE lncRNAs that may play a role in pSS, constructed their regulatory networks, and attempted to identify the key lncRNAs that affect the pathogenesis of pSS.

In this study, GO and KEGG pathway functional enrichment analysis of DE mRNA showed that biological processes were significantly enriched in the chemokine-mediated signaling pathway, immune response, inflammatory response, cell adhesion, and type I interferon signaling pathway. These biological processes have been reported relating to the pathogenesis of pSS [11]. Type I interferon signaling has been found in the pathogenesis of many rheumatic diseases, as well as in pSS. Type I interferon signature genes (ISGs), such as IRF5 and STAT4 [18, 19], have been considered as susceptibility factors in pSS. In patients with pSS, peripheral blood type 1 interferon signature correlates with the existing anti-SSA/Ro antibodies, higher B cell-activating factor (BAFF) gene expression in monocytes, and clinical disease activity [20, 21]. IFI44 and IFI44L, two molecules of ISGs, have been identified as two of the hob genes that may be involved in the development of pSS [12, 13]. In this study, several ISGs, including IFIT1B, IFI27, IFI44, IFI44L and IFI6, were found to be significantly up-regulated in the PBMCs of pSS patients, which suggested the activation of type 1 interferon signaling in these patients.

Another significantly up-regulated gene in this study was EPSTI1. Recent studies have recognized elevated EPSTI1 levels as a promoter for B cell activation by activating TLR9 signaling and considered it a key factor for pSS pathogenesis [14]. In the present study, through the analysis of the PPI network of DE mRNAs, we identified several hub genes, including IL6, IL10, and CXCL8, which are important genes in autoimmune diseases and inflammatory pathways. Based on these findings, we believe that there is an active autoimmune inflammatory response in patients with pSS.

From the top 15 up-regulated and down-regulated DE lncRNAs, we chose four lncRNAs: GABPB1-AS1, PSMA3-AS1, LINC00847 and SNHG1, and constructed their ceRNA network. Further analysis showed that the expression level of two up-regulated lncRNAs (GABPB1-AS1 and PSMA3-AS1) were positively correlated with ISGs (IFI44, IFI44L, IFI6), as well as EPSTI1. These two lncRNAs were validated using real-time PCR in an independent cohort that included 30 pSS patients and 15 healthy controls. The results indicated that GABPB1-AS1 and PSMA3-AS1 were significantly up-regulated in patients with pSS. LncRNA GABPB1-AS1 has been shown to play an important role in cancer pathogenesis through a mechanism that controls gene expression. For example, GABPB1-AS1 is an lncRNA associated with autophagy and may play a key role in glioma biology [22]. GABPB1-AS1 was significantly up-regulated in HPV16-positive cervical cancer tissues and associated with a poor prognosis in these patients [23]. It has been shown that GABPB1-AS1 regulates cellular oxidative stress in liver tumor cells and may be an attractive therapeutic target for hepatocellular carcinoma [24]. GABPB1-AS1 inhibited renal cell carcinoma growth and played a tumor suppressor role [25]. Therefore, we predicted that GABPB1-AS1 might participate in the pathogenesis of pSS by affecting type 1 interferon signaling.

BAFF was discovered to be essential in pSS pathogenesis by promoting the activation and proliferation of B cells and was reported to be significantly up-regulated in PMBCs of pSS patients [26, 27]. According to the prediction in the StarBase database, GABPB1-AS1 interacts with miR-16-5p and miR-155-5p. MiR-16 has been shown to be involved in the occurrence of several autoimmune diseases, as well as in pSS, by controlling cytokine expression [28]. miR-155 is an important regulator of B cell activation through the TNF-α/BAFF/CD19 signaling pathway [29, 30]. However, miRNAs can act as a “sponge” by lncRNAs to achieve regulatory functions. Therefore, we deduced that GABPB1-AS1 may regulate the activation of B cells by interacting with miR-16 or miR-155. However, this regulatory relationship or other potential regulatory mechanisms underlying differentially expressed lncRNAs affecting pSS pathogenesis needs further experimental verification.

The lncRNA and mRNA expression profile in this study was quite different from the 2 previous similar studies [10, 11]. Considering that gene expression analysis only reflect a certain stage of the disease, samples from different pSS patients may vary greatly. In this study, the four patients in the dicovery cohort had very similar clinical features. All the pSS patients we recruited hadn’t been given immunosuppressive treatment previously and 80% patients were diagnosed pSS with the result of labial salivary gland biopsies. Compared of 11 DE LncRNAs abtained in previous studies, only NRIR was similarly up-regulated (fold change = 7.1, *p* = 0.002) in this study based on RNA sequencing and other 10 lncRNAs showed no differential expression (Table S4). However, there is some overlap in the expression pattern of gene pathways. Type I interferon signaling pathway was significantly enriched in all 3 studies, indicating its importance in the pathogenesis of pSS. We used real-time PCR to verify the two significantly up-regulated lncRNAs in a relatively large cohort to ensure the reliability of the results. Interestingly, we found the expression level of GABPB-AS1 was significantly positively correlated with the percentage of B cells. The possible mechanism of GABPB-AS1 involved in the activation of B cell was further analyzed, which laid foundation for the next research.

## Conclusion

In this study, we identified two significantly up-regulated lncRNAs, GABPB1-AS1 and PSMA3-AS1, in pSS patients compared to health controls. GABPB1-AS1 was found to be up-regulated 3.0-fold in pSS patients, and its expression level is positively correlated with the percentage of B cells and IgG levels. GABPB1-AS1 is predicted to play an important role in the pathogenesis of pSS and may be a promising biological marker in the disease.

This study add new evidences on the importance of lncRNAs in the pathogenesis of pSS and may offer promising clues for novel diagnosis method and therapeutic target to the disease.

## Methods

### Patients

Thirty female patients diagnosed with pSS and 15 sex- and age-matched healthy persons were recruited from the Second Affiliated Hospital of Zhejiang University. The enrolled pSS patients met the diagnostic criteria of the 2016 American College of Rheumatology (ACR)–European League against Rheumatism (EULAR). The severity of dryness was evaluated using the patient-reported visual analogue scale (VAS). Results of laboratory tests and salivary gland biopsy were collected, as well as systemic involvement of the patients. The disease activity of pSS patients was evaluated using ESSDAI. The enrolled patients did not have immunosuppressive treatment prior to blood specimen collection. Peripheral venous blood samples (5 mL) were extracted from each participant using BD Vacutainer K2EDTA tubes, and PBMCs were immediately isolated by Ficoll-Hypaque density gradient separation. Trizol was added to PBMC samples immediately, then cells were lysed and subjected to total RNA extraction. This mixture was stored in a − 80 °C refrigerator until RNA extraction. The discovery cohort was composed of 4 pSS patients and 4 healthy controls, and the independent validation cohort was composed of 30 pSS patients and 15 healthy controls.

Prior to the blood sample collection, written informed consent was obtained from the participants. The protocols used in this study were performed following the principles of the Helsinki declaration and were approved by the Ethical Committee.

### RNA extraction and purification

Total RNA was extrated from PBMCs using the miRNeasy Mini Kit (Qiagen, Germany) following the manufacturer’s instructions. High-purity RNA was obtained using the RNAClean XP Kit (Beckman Coulter, USA) and RNase-Free DNase Set (Qiagen). RIN number of RNA samples was detected on an Agilent Bioanalyzer 2100 (Agilent Technologies, USA) and NanoDrop ND-2000 spectrophotometer to evaluate RNA integrity. Samples with qualified measurements (2100 RIN ≥7.0, 28S/18S ≥0.7) were included in the subsequent transcriptome sequencing.

### Transcriptome sequencing

Eight RNA samples from the discovery cohort were used for transcrptome sequencing. Sequencing RNA sample library construction was performed through a series of steps, including rRNA removal, fragmenting, first-strand cDNA synthesis, second-strand cDNA synthesis, terminal repair, addition of 3′ terminal A overhang, connection, and enrichment. The concentration was determined using a Qubit®2.0 Fluorometer and library size on Agilent 4200.

Cluster generation and first-to-sequencing primer hybridization were conducted on the cBot with the Illumina sequencer following the procedures acquired from the cBot instruction. Sequencing reagents were prepared according to the Illumina user guide, and flow cells carrying clusters were used for double-ended sequencing by the paired-end program. Data collection software of Illumina was controlled and analyzed in real-time the sequencing process.

High throughput sequencing (Illumina Hiseq 2000/2500 and Miseq) was used to complete the cDNA sequencing. Raw sequencing reads were filtered to obtain clean reads. The spliced mapping algorithm of HISAT2 (version 2.0.4) was used for genome mapping of the clean reads, using a genome version of hg38 (ftp://ftp.ensembl.org/pub/release-90/fasta/homo_sapiens/dna/Homo_sapiens.GRCh38.dna.toplevel.fa.gz). The number of reads was converted to Fragments Per Kilobase of transcript per Million mapped reads (FPKM) values using Perl scripts after fragment counting (StringTie, version 1. 3.0) and normalization (trimmed mean of M values, TMM). Transcriptome sequencing read depths and mapping efficiency was shown in Table S1. Finally, 38,096 lncRNAs and 50,869 mRNAs were investigated.

After eliminating the low-expressed genes in the data (genes whose raw count was 0 in more than 75% samples), genes with a different expression between pSS samples and health samples were analyzed by edgeR. Meanwhile, we calculated the differential expression multiple (fold-change) based on the FPKM value. LncRNA and mRNA expression with a fold-change > 2 and a *p*-value less than 0.05 were considered to be differentially expressed genes.

### Bioinformatics analysis

GO and KEGG functional enrichment analysis of mRNAs with different expression levels was accomplished using the DAVID online tool (https://david.ncifcrf.gov/; version 6.8). The results include Biological Process, Cellular Component, and Molecular Function. The Fisher test was used, and the enrichment threshold was set at *p*-value < 0.05.

The PPI of differentially expressed genes was obtained using the STRING (http://www.string-db.org/; version 10.0) database. PPI networks of differentially expressed genes were constructed using Cytoscape software (version 3.2.0). In the PPI networks, functional modules were identified by the MCODE plug-in of Cytoscape software, and finally, biologically meaningful protein complexes were obtained. Parameters were as follows:include loops: false degree cutoff: 10, node score cutoff: 0.2, cut: true, FF: false, K-Core: 2, max. Depth from seed: 100. GO and KEGG pathway enrichment analyses of genes in the modules were carried out.

lncRNA target genes were predicted using Trans and Cis regulation. Cytoscape software was used to construct the regulation network of top 15 up-regulation and down-regulation DE lncRNAs. MiRNAs and mRNAs that have interaction relationship with these DE lncRNAs were predicted in the StarBase Database (http://starbase.sysu.edu.cn/; version 2.0). The miRNA targets of DE mRNAs were predicted using the MiRDB Database (http://www.mirdb.org/). PSS related miRNAs were obtained from the HMDD Database (http://www.cuilab.cn/hmdd; version 3.2). Combined the interaction relationship acquired from the above two databases and the 21 pSS related miRNAs, the lncRNA-miRNA-mRNA network, or the ceRNA network, was constructed by cytoscape software. Then, the co-expression relationship between the key lncRNAs in the ceRNA network and the 15 most up-regulated and down-regulated DE mRNAs was analyzed by Spearman’s test.

### Real-time PCR

The lncRNAs screened for abnormal expression were validated using real-time PCR in all the 45 extracted RNA samples. The lncRNAs we screened were based on the following criteria: (1) fold change ≥4, (2) average FPKM of the up-regulated groop ≥5, (3) without repeated sequences of mRNA, and (4) lncRNAs without information in databases were excluded.

The purified total RNA was reverse transcribed into cDNA using the ReverTra Ace qPCR Kit (TOYOBO, FSQ-101). The obtained cDNA sample was subjected to real-time quantitative PCR using the Power SYBR Green PCR Master Mix (ABI, USA). A QuantStudio 5 Real-Time PCR System (ABI) was used for the PCR procedures. LncRNA expression was measured and normalized to the mean expression of the housekeeping gene: GAPDH (Table S2). The relative fold change of each sample was calculated in relation to the ∆Ct of a random unstimulated sample (reference) in the health control group according to the formula: fold change = 2^−∆∆Ct^, where ∆∆Ct = ∆Ct sample-∆Ct reference.

### Statistical analyses

GraphPad Prism 7.0 version was used for statistical analysis of the data. Data are represented as fold changes relative to healthy controls. Correlation analysis was performed by Spearman’s test. An analysis based on the two-tailed unpaired t-test or Mann-Whitney U test was performed to evaluate differential expression of genes between groups. The significant difference was the *p*-value of less than 0.05. False discovery correction was not used to adjust *p*-value because of the small number of DE lncRNAs and mRNAs.

## Supplementary Information

**Additional file 1: Table S1**. Transcriptome sequencing read depths and mapping efficiency; **Table S2**. The selected lncRNAs and the housekeeping gene (GAPDH) and their primers. **Tables S3**. Correlation analysis between expression levels of GABPB1-AS1 and PSMA3-AS1 and some clinical parameters. **Table S4**. Comparison of differentially expressed LncRNAs abtained in previous similar studies with this study.

## Data Availability

The datasets generated and analysed during the current study are available in the GEO database, https://www.ncbi.nlm.nih.gov/geo/query/acc.cgi?acc=GSE164885.

## Abbreviations

pSS: Primary Sjögren’s syndrome
lncRNA: Long non-coding RNA
PBMC: Peripheral blood mononuclear cell
PPI: Protein interaction relationship
ceRNA: Competitive endogenous RNA
miRNA: Short-chain non-coding RNA
IFN-γ: Interferon-γ
MMP: Promoting matrix metalloproteinase
ESSDAI: Eular Sjogren’s syndrome disease activity index
DE: Differential expression
ISG: Interferon signature gene
BAFF: B cell-activating factor
ACR: American College of Rheumatology
EULAR: European League against Rheumatism
VAS: Visual analogue scale
FPKM: Fragments Per Kilobase of transcript per Million mapped reads
