# Supplementary material for: Differential long non-coding RNA expression profile and function analysis in primary Sjogren’s syndrome
Source: BMC Immunol. 2021 Jul 20;22:47. doi: 10.1186/s12865-021-00439-3 (PMC8293522; doi:10.1186/s12865-021-00439-3)
Supplement: Supplementary file 1 — Additional file 1: Table S1. Transcriptome sequencing read depths and mapping efficiency; Table S2. The selected lncRNAs and the housekeeping gene (GAPDH) and their primers. Tables S3. Correlation analysis between expression levels of GABPB1-AS1 and PSMA3-AS1 and some clinical parameters. Table S4. Comparison of differentially expressed LncRNAs abtained in previous similar studies with this study. [file 12865_2021_439_MOESM1_ESM.docx]

Table S1. Transcriptome sequencing read depths and mapping efficiency

| **Sample** | **All reads** | **Mapped reads** | **Mapping ratio** |
| --- | --- | --- | --- |
| PSS1 | 62,854,068 | 61,834,240 | 98.4% |
| PSS2 | 68,600,470 | 67,284,472 | 98.1% |
| PSS3 | 72,932,406 | 71,685,865 | 98.3% |
| PSS4 | 81,658,122 | 80,317,193 | 98.4% |
| HC1 | 73,066,362 | 71,813,038 | 98.3% |
| HC2 | 63,469,462 | 62,455,161 | 98.4% |
| HC3 | 62,586,114 | 61,551,218 | 98.3% |
| HC4 | 69,622,918 | 68,473,251 | 98.3% |

PSS: primary Sjogren syndrome; HC: health control

Table S2. The selected lncRNAs and the housekeeping gene (GAPDH) and their primers

| **Gene name** | **LncRNA_id** | **Primer name** | **Primer sequence(5' to 3')** |
| --- | --- | --- | --- |
| PSMA3-AS1 | NONHSAT037119.2 | NONHSAT037119.2-F | GCTCTGGTTTCTGTTCCCTGTT |
|  |  | NONHSAT037119.2-R | ACATTCAGTCCATGGCTGCTT |
| GABPB1-AS1 | NONHSAT042411.2 | NONHSAT042411.2-F | TGAAAATGACTGAGGGAGATAGATGT |
|  |  | NONHSAT042411.2-R | CCTCCTAGCTGCTCCACCAA |
| GAPDH | / | Gapdh-F | TGACTTCAACAGCGACACCCA |
|  |  | Gapdh-R | CACCCTGTTGCTGTAGCCAAA |

Tables S3. Correlation analysis between expression levels of GABPB1-AS1 and PSMA3-AS1 and some clinical parameters

| **Clinical parameters** | **GABPB1-AS1** | | **PSMA3-AS1** | |
| --- | --- | --- | --- | --- |
|  | **Pearson r** | **P value** | **Pearson r** | **P value** |
| IgG | 0.4702 | 0.0087** | 0.2725 | 0.1452 |
| B cell | 0.7883 | < 0.0001** | 0.3273 | 0.0775 |
| C3 | 0.1394 | 0.5361 | 0.2246 | 0.315 |
| C4 | 0.01636 | 0.9424 | 0.05919 | 0.7936 |
| ESR | 0.06578 | 0.7712 | 0.09482 | 0.6747 |
| RF | 0.2359 | 0.2907 | 0.2101 | 0.3481 |
| ESSDAI | -0.2479 | 0.2659 | 0.09777 | 0.6651 |

IgG: immunoglobulin G; C3/C4: complement 3/4; ESR: erythrocyte sedimentation rate; RF: rheumatoid factor; ESSDAI: Eular Sjogren’s syndrome disease activity index; **: P ＜0.01

Table S4. Comparison of differentially expressed LncRNAs abtained in previous similar studies with this study

| **LncRNAs** | **Previous studies** | | **This study** | |
| --- | --- | --- | --- | --- |
|  | **Fold change** | **P value** | **Fold change** | **P value** |
| NRIR | 5.3 | 0.003** | 7.1 | 0.002** |
| BISPR | 2.2 | 0.012* | 1.4 | 0.633 |
| CYTOR | 1.6 | 0.002** | / | / |
| LINC00426 | -3.8 | 0.001** | 1.4 | 0.853 |
| TPTEP1-202 | -2.9 | 0.002** | / | / |
| LINC00657 | 1.8 | 0.001** | -1.1 | 0.595 |
| LINC00511 | -2.0 | 0.008** | / | / |
| CTD-2020K17.1 | 2.7 | ＜0.001** | 1.0 | 1.000 |
| RP11-169K16.9 | 1.7 | 0.008** | / | / |
| RP11-214O1.2 | -2.1 | 0.010* | -1.4 | 0.143 |
| RP11-372K14.2 | -2.1 | 0.004** | 2.0 | 0.103 |

*: P＜0.05；**: P ＜0.01; / : not detected
